# Exploratory study of introducing HPC to non-ICT researchers: institutional strategy is possibly needed for widespread adaption

**DOI:** 10.1007/s11227-020-03438-0

**Published:** 2020-09-28

**Authors:** Bence Ferdinandy, Ángel Manuel Guerrero-Higueras, Éva Verderber, Francisco Javier Rodríguez-Lera, Ádám Miklósi

**Affiliations:** 1 MTA-ELTE Comparative Ethology Research Group, Budapest, Hungary; 2 Robotics Group, Department of Mechanical, Computer Science, and Aerospace Engineering, University of León, León, Spain; 3 Faculty of Education and Psychology, Eötvös Loránd University, Budapest, Hungary; 4 Department of Ethology, Eötvös Loránd University, Budapest, Hungary

**Keywords:** Supercomputing, HPC, Education, Professional development

## Abstract

Machine learning algorithms are becoming more and more useful in many fields of science, including many areas where computational methods are rarely used. High-performance Computing (HPC) is the most powerful solution to get the best results using these algorithms. HPC requires various skills to use. Acquiring this knowledge might be intimidating and take a long time for a researcher with small or no background in information and communications technologies (ICTs), even if the benefits of such knowledge is evident for the researcher. In this work, we aim to assess how a specific method of introducing HPC to such researchers enables them to start using HPC. We gave talks to two groups of non-ICT researchers that introduced basic concepts focusing on the necessary practical steps needed to use HPC on a specific cluster. We also offered hands-on trainings for one of the groups which aimed to guide participants through the first steps of using HPC. Participants filled out questionnaires partly based on Kirkpatrick’s training evaluation model before and after the talk, and after the hands-on training. We found that the talk increased participants’ self-reported likelihood of using HPC in their future research, but this was not significant for the group where participation was voluntary. On the contrary, very few researchers participated in the hands-on training, and for these participants neither the talk, nor the hands-on training changed their self-reported likelihood of using HPC in their future research. We argue that our findings show that academia and researchers would benefit from an environment that not only expects researchers to train themselves, but provides structural support for acquiring new skills.

**Electronic supplementary material:**

The online version of this article (10.1007/s11227-020-03438-0) contains supplementary material, which is available to authorised users.

## Introduction

In today’s digitally driven world, science is increasingly dependent on advanced technology. Facing certain problems in areas such as healthcare and life sciences, astrophysics, meteorology, climatology, or artificial intelligence requires enormous amounts of computational power. Information society requires better-prepared professionals in order to face these problems. These professionals should acquire several skills in order to manage High-performance Computing (HPC) environments. The acquisition of these skills is especially relevant because HPC environments require specific technologies to work with.

However, there are many fields where although HPC would be of great use that are practised by professionals, who have little to no training in Information and Communications Technologies (ICT), and would thus quite likely think that HPC is too complicated for them to use. For example in ethology, the possibility of animal-borne sensors and ever smaller cameras with more and more digital storage creates the possibility of recording unprecedented amount of data about animal behaviour. Supercomputers not only help in the analysis of this data (e.g. [7, 9]), but also in theoretical works to run large scale simulations (e.g. [8]). Unfortunately, ethologists themselves are rarely equipped with the skills required to write or sometimes even to run these software. Thus these works are quite often done by people from outside the field, although the field would very much profit if ethologists themselves were better trained in this regard.

Institutions are devoting a lot of effort and investing great amount of money to promote and foster HPC facilities, but less effort is placed into teaching HPC and assessing whether some HPC basics have been acquired by the people encouraged to use them. It is thus necessary to work at developing the system of supercomputing education in order to prepare the professionals for the current reality of HPC [20].

There are some research in the literature about education in HPC. For instance, Harrel et al. detail the design of an online course focusing on parallel solutions, presenting common HPC use cases and the strategies for parallelising them. They present the Student Cluster Competition (SCC) which was created as an educational tool to immerse undergraduates in HPC [11]. Neumann et al. [17] address the issue of teamwork from the perspective of HPC. Antonov et al. [5] discuss the experience of teaching supercomputer disciplines to students specialising in Computational Mathematics since they have a high probability of becoming future developers and users of complex supercomputing applications and systems.

However, it is important to point out that most research on education in HPC is about professionals or undergraduates of Information and Communications Technologies (ICT) and that most users of HPC also come from these fields or a few select fields that are close to ICT (mathematics, physics). Thus, no research has yet been conducted about teaching to use HPC to researchers who have little to no background in ICT and are the first time in a situation where they need to address a problem using supercomputing. Using supercomputing requires various skills, at a minimum, using the command line on a remote Linux computer, but in practice, minimal knowledge in shell scripting is also needed. Although the need for acquiring new skills is very important to researchers of all fields, transitioning from a local computer’s graphical interface to a remote computer’s terminal is one that needs a larger cognitive shift then most new skills, and as such could be considered quite daunting by researchers, compared to other types of new skills. Nonetheless, problems associated with learning HPC can be placed into a broader academic and higher education context.

In the last few decades, higher education is facing new tendencies such as massification, displacement from teacher and teaching centred approach to student and learning centred approach, internationalisation and the growing importance of new digital technologies. Globalisation and the increase of the knowledge economy has had a huge impact on research in higher education institutions. It has changed the definition of efficiency, the foundations of the financial background of institutions, and importance of cooperations between academia and industrial sectors [16]. Increasing competition is taking place between universities, research groups, and between researchers. Research capacity of a university is related to individuals continuing professional development which is based on learning new competencies, especially from the field of ICT, which can provide their research efficiency and ensure their competitive edge in the long run.

Universities play a vital role in building national research capacity and academics play a vital role in knowledge creation and knowledge transfer processes across a country, and between scientific fields [10].

Building research capacity has an individual level, with individual training and career development, and an institutional level, with institutional programmes. In the first case, the main focus is on the level of individual, and research capacity is primarily based on the expertise of a researcher, while in the second case, is on the level of organisation [24].

Continuing Professional Development (CPD) becomes the core element of every profession in order to adapt the ever-growing competitiveness. CPD is a process by which individuals take control of their own learning and development based on individuals’ autonomy and competencies and needs for responsibility of their own professional and personal growth [19, 21, 23]). CPD among academics usually focuses on improving pedagogical skills rather than researcher roles and skills or both at the same time.

Researchers’ competencies are directly related to the effectiveness of investments in research and development, and the prestige of the higher education institution at which they work; however, academics rarely participate in CPD activities, and even if they do it usually not systematically planned [12].

Skill-needs of researchers are evolving, thus CPD and trainings for improving research skills are more and more valued. Nowadays researchers face new academic pathways and expanded challenges. They need skills that will allow them to reach better technological and innovative performance. Formal trainings play a part in keep updating and building on researchers’ existing skills related to their core, and broader research skills, as well [2, 22].

Even though academics’ efficiency is based on their personal scientometric indexes, the organisational level is highly critical because the support systems of research groups, departments, faculties etc., have a huge impact on individual achievements. Even though literature is more focused on CPD as pedagogical capacity, similar obstacles could be described as research capacity of academics. Such as:

- academics’ unwillingness to move away from traditional research methods/practices
- lack of time for CPD among university staff, and
- lack of financial, organisational, and institutional capacity to develop effective CPD schemes at the university level [12].

In the current study, we investigate the effectiveness of learning activities introducing HPC aimed at researchers who do not have an ICT background. Our approach was to focus on the necessary practical steps needed to use HPC in order to show these are not necessarily complicated and giving practical examples to where HPC can be applied. Thus the novelty of our study is twofold: i) we investigate HPC education in non-ITC trained people, and ii) we investigate gaining skill in research at a higher educational institution.

## Materials and methods

### Outline

We gave talks to two groups which included a general introduction to HPC, details of the actual process of using an HPC system and examples of practical cases where it can be used. One session (Group A) was held in September of 2019 and a hands-on training was offered for participants following the talk, which was held over the course of 2 weeks following a week after the talk, focusing heavily on the use of a domain-specific tool. The other session (Group B) was held in June of 2020, which was held online and the organisation of a hands-on training was not possible due to the COVID-19 pandemic.

Three different questionnaires were taken to be used for the analysis of subjects’ demography, reactions and expectations regarding supercomputing and the specific learning activities. The questionnaires were provided to participants via Google Forms. The first one, $Q_1$, gathered demographics and established a baseline of attitude and previous knowledge and was filled out before the introductory talk. The second questionnaire, $Q_2$, was filled right after the introductory talk, while $Q_3$, was filled after the hands-on course by those participating in it. Questionnaires were anonymous, but participants were asked to generate a unique, 5 character long ID for us to be able to connect individuals across questionnaires. To generate the characters of the ID, the participants received the following instructions: “(1) The first letter of your birth month written in English. (2) The last digit of the day of your birth. (3) The first letter of your mother’s maiden last name. (4) The second letter of your mother’s first name. (5) The last letter of your father’s first name.” Although this ID is not guaranteed to be unique in a mathematical sense, but is highly unlikely to generate duplicates within small groups of people and participants can regenerate it anytime without the need to remember them, thus is an effective way to generate anonymous ID-s. For further details, we provide the entire dataset, along with the original questions received by participants in the Supplementary Materials.

### Demography

Group A subjects were recruited from the Department of Ethology, Eötvös Loránd University, Hungary and closely associated research groups which operate in the same location. Participation in the introductory talk was semi-compulsory it was held during the usual weekly meeting of the Department of Ethology, which is not explicitly compulsory, but people are expected to attend regularly. The hands-on course was entirely voluntary and was scheduled according to the availability of volunteers over a 2-week period. Both were advertised beforehand through the Department’s e-mailing list.

In total 29 people attended the introductory talk, but only 25 filled out both $Q_1$ and $Q_2$, so only these 25 were kept for later analysis (16 women, 9 men; 14 Ph.D. students, 6 postdocs and 5 senior researchers; age: 35.28 ± SD 8.49).

In total 5 people attended the hands-on course, but one of the participants did not attend the introductory talk, and was thus excluded (2 women, 2 men; 2 Ph.D. students, 2 postdocs; age: 31.00 ± SD 3.92).

Group B subjects were recruited from the University of León, Spain mainly from the faculties of Veterinary Sciences, Biological and Environmental Sciences, and Economics. Participants were reached through snowball emails and participation was voluntary and was completely online. As mentioned before, due to COVID-19, it was not possible to hold a hands-on training.

In total 26 people attended the introductory talk, but only 19 filled out both $Q_1$ and $Q_2$, so only these 19 were kept for later analysis (10 women, 9 men; 1 student, 7 PhD students, 1 postdoc and 10 senior researchers; age: 39.10 ± SD 11.45).

### Background of participants

**Fig. 1:**
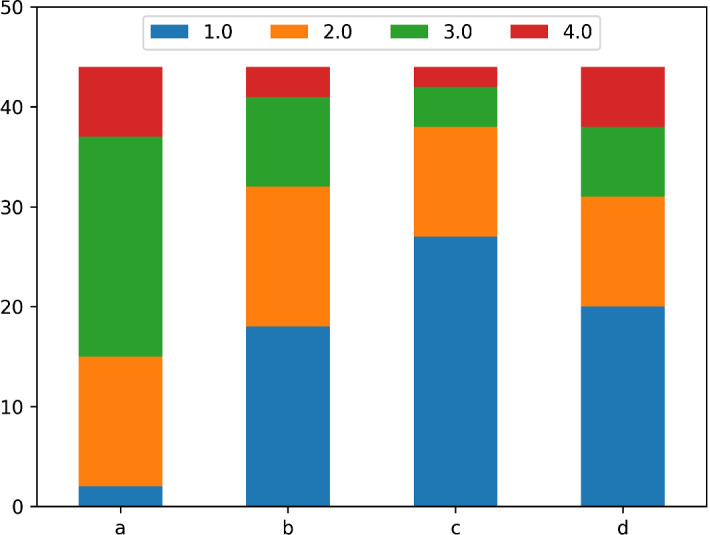
Background of participants of the talk. Questions were rated from 1 (lowest skill or confidence) to 4 (highest skill or confidence), median values are reported in parenthesis. **a** Rate your computer skills (3). **b** Rate your programming skills (2). **c** Rate your confidence in using supercomputing (1). **d** Expert scoring of the answers to: describe what supercomputing (high-performance computing) is (3)

Participants’ background was assessed in $Q_1$ with self-evaluation questions and a question “Describe what supercomputing (high-performance computing) is.” which was later scored by 5 experts (researchers at the University of León, Spain, with considerable experience with HPC) on a 1 to 4 scale, to quantify how accurate the response was (the median score of the 6 experts were used in further analysis). There is reasonable correlation (Spearman’s $r = 0.61, p < 0.001$) between this score and participants’ confidence level in using supercomputing. See Fig. 1 for questions and the scoring. The questions could be answered on an ordinal scale from 1 (lowest skill or confidence) to 4 (highest skill or confidence), thus we only report the median values.

Participants reported good (median of 3) computer skills, but little knowledge in programming (median of 3) and no knowledge in supercomputing (median of 1).

Overall, participants’ level of expertise is very low with regards to supercomputing, and based on their self-assessments, their programming skills are also low, but their ability to handle computers is adequate on average, thus they fit the scope of the current study.

### Training evaluation: Kirkpatrick’s four levels of evaluation

Evaluation is the key to know the impact of learning programs, courses, trainings, etc. Workplace learning opportunities can have a dramatic impact on business performance by changes in specific knowledge, skills, attitudes or behaviours of employees. In higher education, this can be described as academics’ growing efficiency in teaching and research performance. These learning opportunities have the potential to be really transformative for the effectiveness, especially when they are based on evidence and data-driven decisions. Training evaluation is the process of information and data collecting systematically which is planned along with the training plan, based on the planning objectives and goals the organisation wanted to obtain [13].

Kirkpatrick’s model [13, 14] is one of the most well-known learning evaluation model which is implemented well in practice. Its four levels are:

1. reaction
2. learning
3. behaviour
4. results

Reaction level is focused on the participants’ thoughts, feelings, and satisfaction about the training. It describes whether the information, the process of knowledge sharing was effective and appreciated. It helps to improve the training, to identify topics and areas that are missing from the training, and its perceived value and transferability to the workplace. It is captured by surveys following the training.

Learning level is focused on what the participants have learned, i.e. the resulting increase in knowledge or capability. It is captured by assessments or tests before and after the training to describe a difference.

Behaviour level is focused on how the participants change their behaviour based on the training received, so the main focus is training effectiveness rather than training evaluation. Evaluation of implementation and application is vital and challenging at the same time. It is captured by surveying learners after the training, when they have returned to their work. We have to empathise that behaviour can only change if the conditions are favourable, supporting is reachable from the organisation, and encouraged by leaders.

Results level describes the final results of training, and is focused on the outcomes that the organisation has determined to be good for the business (teaching and/or research), and good for the participants. It can only really be measured by looking at business data (or other measures of relevant output, e.g. number and quality of research papers) relating to the training.

In Table 1, we show the above levels correspond to the setting and aims of our learning activities.

**Table 1:** Kirkpatrick’s four levels of evaluation model applied to study goals [13]

Level	Description
Level 1: reaction	The degree to which participants find the training favourable, engaging and relevant to their research
Level 2: learning	The degree to which participants acquire the intended knowledge, skills, attitude, confidence and commitment based on their participation in the training
Level 3: behaviour	The degree to which participants apply what they learned during training during their normal research routines
Level 4: results	The degree to which targeted program outcomes occur and contribute to better research capabilities on departmental level

### HPC environment

An HPC environment has three basic components: (1) an HPC facility, (2) a resource manager to manage the accesses to the HPC facility, and (3) one or more parallel frameworks to work with.

For this work, researchers were introduced to a specific HPC environment described below. During the introductory talk this facility was used as an example, and during the hands-on course, participants were granted access to this facility.

Caléndula is the cluster of Supercomputación Castilla y León (SCAyLE). SCAyLE has several calculation clusters with different computer technology architectures. Participants accessed a cluster dedicated to teaching [1].

Calendula uses SLURM for resource management. It is a free and open-source job scheduler for Linux and Unix-like kernels [25]. It is used by many of the world’s supercomputers and computer clusters. Slurm provides three basic services. First, it allocates exclusive and/or non-exclusive access to resources (computer nodes) to users for some duration of time so they can perform work. It also provides a framework for starting, executing, and monitoring work (typically a parallel job such as MPI) on a set of allocated nodes. Finally it arbitrates contention for resources by managing a queue of pending jobs. Slurm uses a best fit algorithm in order to optimise locality of task assignments on parallel computers [18].

### Domain-specific tool for hands-on training

The hands-on training with only for Group A due to COVID-19, thus the domain specific tool was aimed at ethologists.

Ethology studies animal behaviour by observing the animal behaviour in various contexts and coding the observed behaviour according to the relevant study questions. In the beginning, this was done in situ during observation, but in modern times the typical routine is to make video recordings of the behaviour which is analysed later to get quantitative results (examples with various taxa are, e.g. experiments with dogs [3], capuchin monkeys [4], cleaner fish [6], and zebra finches [15]). The possibility of recordings opened the possibility for obtaining a wealth of data, but due to lack of tools, analysis is mostly done with human effort.

In order to ease the burden on human analysts, the application LabDogTracker has been developed to track the movement of dogs and humans within the lab of the Department of Ethology, as these are the two most common subjects at the department. Until this study, none of the staff have actually seen or used it before (except for the developer, who is also a co-author of this paper). The application relies on using a pre-trained neural network to find the location of dogs on the images of five cameras mounted on the ceiling of the lab, with multiple cameras’ field of view covering any given area in the lab. The coordinates measured on the images are then mapped to the physical space of the lab and later merged into paths. The paths are exported into text files, which can be later used to answer simple ethological questions. For example, many experiments require an answer to question such as: how much time did the dog spend around their owner, or how much time the dog spent in a specific place, or how fast the dog moved around.

The computationally most expensive part of the application is the video analysis. Using a PC with an average GPU analysing a 6 min long experiment requires time on the order of a full day. For a typical study at the Department of Ethology there will be around 30–50 measurements, which can easily be longer than 6 min, thus analysing an entire study on one’s own PC could take several months. As such running the LabDogTracker in an HPC environment would be highly useful for the researchers.

### Participants’ reaction to the learning activities

**Fig. 2:**
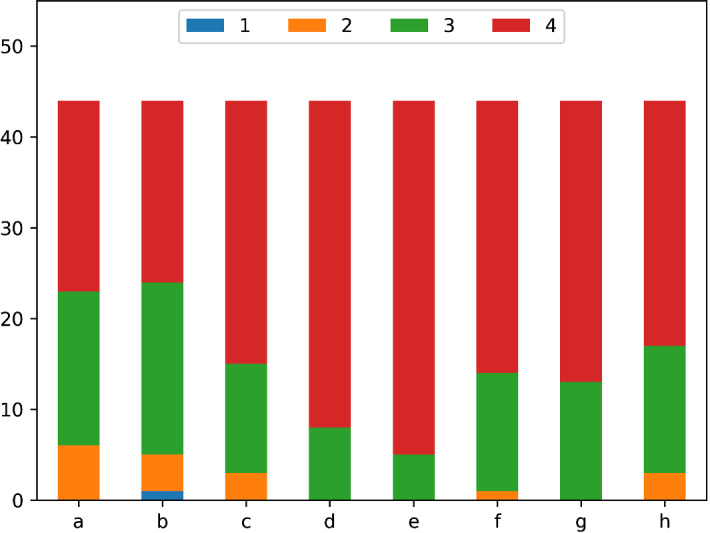
Level 1 evaluation of the introductory talks. Questions were rated from 1 (strongly disagree) to 4 (strongly agree), median values are reported in parenthesis. Mann–Whitney-*U* tests were carried out to test for differences between the two talks. The only difference was found in responsiveness (**d**), where the online lecture was found to be slightly less responsive ($\Delta m = 0.42$, $p < 0.001$). **a** The topics presented were what you expected of the presentation (3). **b** The presentation met your needs (3). **c** The presentation was of adequate length for the topics presented (4). **d** The presenter was responsive to the participants (4). **e** The presenter was knowledgeable in all topics presented (4). **f** The presenter provided adequate visual aids (4). **g** The presenter’s style and delivery was effective (4). **h** Would recommend this presentation to other colleagues (4)

**Fig. 3:**
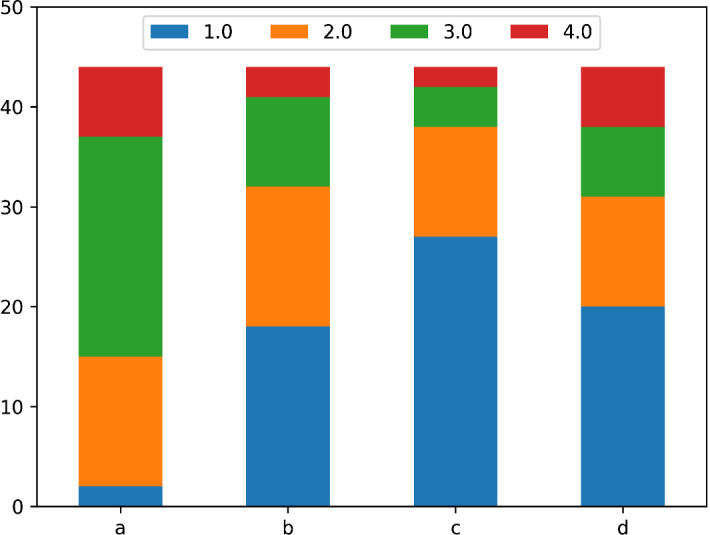
Level 1 evaluation of the hands-on training. Questions were rated from 1 (strongly disagree) to 4 (strongly agree), median values are reported in parenthesis. **a** The topics presented were what you expected of the workshop (4). **b** The workshop met your needs (3.5). **c** The workshop was of adequate length for the topics presented (3.5). **d** The presenter was responsive to the participants (4). **e** The presenter was knowledgeable in all topics presented (4). **f** The presenter provided adequate visual aids (4). **g** The presenter’s style and delivery was effective (4). **h** Would recommend this workshop to other colleagues (4)

Some questions of $Q_2$ and $Q_3$ were aimed at evaluating the learning activities themselves, in order to control for the quality of the talk and hands-on training in our results. The evaluation was based on level 1 of Kirkpatrick’s model, the reaction. See Figs. 2 and 3 for the list of questions and results for both learning activities. The questions could be answered on an ordinal scale from 1 (strongly disagree) to 4 (strongly agree), thus we only report the median values.

For both groups, the majority of the medians of the answers were rated 4 and the lowest median was 3. On any of the questions regarding reactions, only 1 person replied with a score of one once. We used Mann–Whitney-*U* tests to check for differences between the two groups. The only difference we found was that for the question “The presenter was responsive to the participants” Group B, where the talk was delivered online reported on average 0.42 lower score (${p} < 0.001$), which is most likely due to the online nature of the talk.

Overall, the participants’ were happy with the learning activities.

## Results

**Fig. 4:**
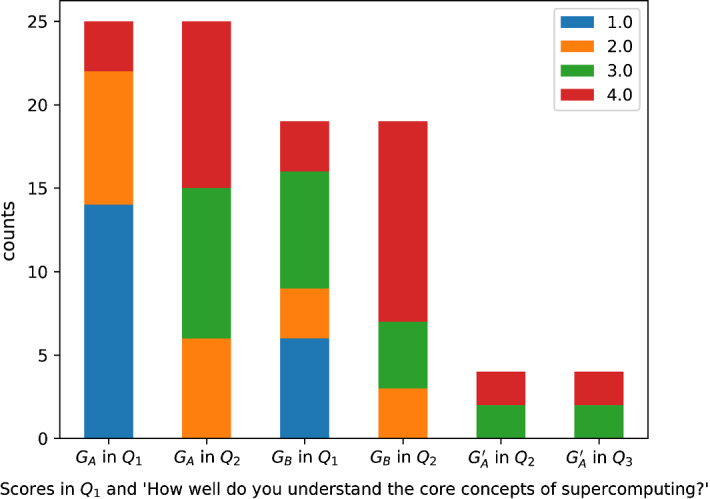
Comparison of expert scoring and self-assessment indicates increase in both groups in understanding after the introductory talk. No change is seen after the hands-on training ($G_A'$ is the subset of people attending the hands-on training)

To assess an increase in knowledge about supercomputing due to the learning activities, we used the expert scoring from $Q_1$ and from $Q_2$ and $Q_3$ the question “How well do you understand the core concepts of supercomputing?” (Fig. 4). Both Group A and B show significant increase in understanding based on Wilcoxon tests (both $p < 0.001$) from $Q_1$ to $Q_2$. We also show on the figure that the hands-on training had no effect on the participants.

**Fig. 5:**
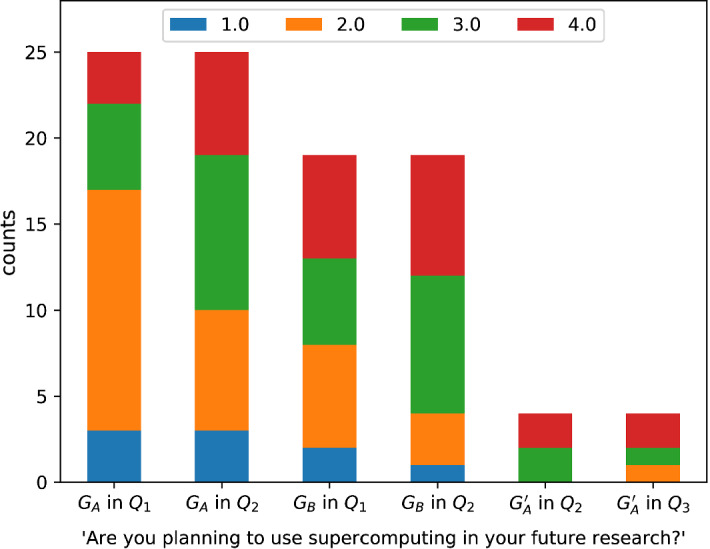
For $G_A$ participation in the talk increased their self-reported probability of using supercomputing in their research (median from 2 to 3), while for $G_B$ there was no significant increase (median of 3). Question was rated on a scale of 1 (certainly not) to 4 (certainly yes)

We asked participants in all questionnaires to self-assess the probability of using supercomputing in their future work (Fig. 5), rated on a scale of 1 (certainly not) to 4 (certainly yes). For $G_A$ the median answer increased from 2 to 3 (Wilcoxon signed-rank test $W = 15, p = 0.048$), while for $G_B$ it stayed the same (median 3, Wilcoxon signed-rank test $W=16.5, p= 0.109$). For the subset $G_A'$ there are not enough values for statistical analysis, we can say that the hands-on training did not really change their attitudes.

## Discussion

The questions regarding participants’ background in $Q_1$ indicate that participants were indeed at the level of expertise we aimed at, that is, they had no prior experience in HPC and had little to moderate experience with ICT, and thus had basically zero experience with the tools needed to use HPC.

In the current study, we had the opportunity to evaluate the learning activities at level 1 (reaction) and partly on level 2 (learning) of Kirkpatrick’s Four Level Evaluation Model (cf. Sect. 2.4). In $Q_2$ and $Q_3$ we asked several questions concerning participants’ reaction to the learning activities, which mostly received a median of maximum score, indicating that participants viewed the learning activities favourably. To properly test the acquired knowledge and skill, we would had to have asked participants to actually try and do some HPC on their own. In case of the introductory talk, this would have not been possible immediately after the lecture, thus we asked for self-evaluations only. Nonetheless, the introductory talk achieved its some of its purpose on level 2 (learning) since self-reported understanding of the subject increased. Contrary to this, the attitude toward HPC only changed in the group where participation was semi-compulsory, but not in the group where it was voluntary.

The effects of the hands-on training can only be analysed anecdotally, since only one of the groups had the chance to attend and turnout was very low, although they reported high interest in attending a hands-on lecture (median of 3 on a 1 to 4 scale). Firstly, participants of the hands-on training did not change their attitude towards using HPC neither after the introductory talk, neither the hands-on training, possibly indicating that their initial attitude were more in line with actual actions they were willing to take. Secondly, the subjective impression of trainer reinforced our a priori assumption, that technicalities (transition from a local graphical interface to a remote command line interface) required a larger cognitive shift, then concepts that were HPC specific (multiple jobs, job handling system, etc.). For example, keeping track of current location (both the current machine, and on a given machine the current path) needed to be revisited multiple times, while submitting jobs was easily mastered.

We argue that (a) the semi-compulsory lecture being more effective and (b) the discrepancy between people interested in and people actually showing up to the hands-on training point to systematic issues with professional development of researchers in higher education. At universities internal (and external) trainings are typically ad-hoc, participation is generally voluntary, and although gaining new skills in itself is regarded as important, time is rarely allocated for it besides other commitments (e.g. conducting experiments, writing manuscripts or teaching classes). Thus, even though learning strategic skills (e.g. HPC) could favourably affect the research capacity of the institution, it is not supported on an institutional level. Since effective harnessing of skills requires a “critical mass” of people with similar or partially overlapping skills, the lack of an institutional strategy leads greatly hinders the ability of the institution to gain new skills and capabilities.

A limitation of our study is the timescale, since all questionnaires were taken during the learning activities, thus any long-term effect could not be observed (e.g. did participants actually start using HPC in their carrier?).

Another limitation is that the hands-on training only involved one group with very few participants, making any claims about the training tenuous.

## Conclusions

In this study, we investigated the effect of giving a talk about HPC, focused on practical issues with usage and offering hands-on training on the attitudes of non-ICT researchers towards the use of HPC. We found that when participation was semi-compulsory the talk about HPC had greater effect, compared to when participation was voluntary. Although due to COVID-19 we could only offer hands-on training to the group where the participation was semi-compulsory, we found that despite large interest reported in the training after the talk, only a handful of people actually attended the training. We argue both are findings show, that unless institutions actively support acquiring new skills like HPC, which requires a major shift from standard practices for non-ICT trained people, researchers will find it hard to commit to learning.

To properly evaluate the effects of the learning activities on all four levels of the Kirkpatrick model, follow-up research needs to be conducted. We plan to reach participants 1 year and 3 years after the original trainings to assess levels 3 and 4 and see whether they have incorporated HPC into their research or not, and whether any research output has been attained with HPC.

Furthermore, since our turnout was low for the hands-on training, our experimental setup will need to be repeated with several other groups of researchers in different institutions, to achieve a higher sample overall sample size.

Since a large part of the scientific endeavour is currently heading towards handling massive amounts of data, institutions and countries who wish to stay ahead must invest not just in HPC infrastructure, but also into enabling researchers to actively apply HPC to their research. Thus we believe more effort needs to be put into developing best practices for integrating learning HPC as part of the CPD of non-ICT researchers.

## Electronic supplementary material

Below is the link to the electronic supplementary material.

Supplementary material 1 (xlsx 13 KB)
